# Conditioning Regimens for Hematopoietic Cell Transplantation in Primary Immunodeficiency

**DOI:** 10.1007/s11882-019-0883-1

**Published:** 2019-11-18

**Authors:** S. H. Lum, M. Hoenig, A. R. Gennery, M. A. Slatter

**Affiliations:** 1 Children’s Haematopoietic Stem Cell Transplant Unit, Great North Children’s Hospital, Newcastle upon Tyne Hospital NHS Foundation Trust, Newcastle upon Tyne, UK; 2 Department of Pediatrics, University Medical Center Ulm, Ulm, Germany; 3 Institute of Cellular Medicine, Newcastle University, Newcastle upon Tyne, UK

**Keywords:** Primary immunodeficiency, Hematopoietic cell transplantation, Reduced toxicity conditioning, HCT outcome, Transplant-related survival

## Abstract

**Purpose of Review:**

Hematopoietic cell transplantation (HCT) is an established curative treatment for children with primary immunodeficiencies. This article reviews the latest developments in conditioning regimens for primary immunodeficiency (PID). It focuses on data regarding transplant outcomes according to newer reduced toxicity conditioning regimens used in HCT for PID.

**Recent Findings:**

Conventional myeloablative conditioning regimens are associated with significant acute toxicities, transplant-related mortality, and late effects such as infertility. Reduced toxicity conditioning regimens have had significant positive impacts on HCT outcome, and there are now well-established strategies in children with PID. Treosulfan has emerged as a promising preparative agent. Use of a peripheral stem cell source has been shown to be associated with better donor chimerism in patients receiving reduced toxicity conditioning. Minimal conditioning regimens using monoclonal antibodies are in clinical trials with promising results thus far.

**Summary:**

Reduced toxicity conditioning has emerged as standard of care for PID and has resulted in improved transplant survival for patients with significant comorbidities.

## Introduction

Primary immunodeficiency (PID) comprises a large, heterogeneous group of disorders that result from defects in immune system development and/or function. Long considered as rare diseases, recent studies show that one in 2000–5000 children younger than 18 years is thought to have a PID. There are now around 350 single-gene inborn errors of immunity and the underlying phenotypes are as diverse as infection, malignancy, allergy, autoimmunity, and autoinflammation. Therefore, presenting features, severity, and age of diagnosis vary immensely. Hematopoietic cell transplantation (HCT) is a well-recognized curative therapy for many of these PIDs. Since the first transplant took place in 1968, utility of HCT was initially limited by high rates of graft failure and transplant-related morbidity and mortality; however, transplant survival and graft outcomes have significantly improved, particularly since 2000 [1, 2]. Many factors have contributed to this improvement including earlier diagnosis, a detailed graft selection hierarchy, superior HLA matching technology, improved methods for graft manipulation, greater availability of grafts, improved supportive care, vigilant infection surveillance and pre-emptive treatment, and more effective antimicrobial therapy. In the modern era, graft engineering, additional cellular therapy, and pharmacokinetic-guided conditioning regimens enable precise personalized transplant care including prescription of graft components, better cell-dosed grafts, and a patient-tailored conditioning regimen [3, 4•, 5••].

Short-term transplant survival outcomes must be carefully distinguished from long-term disease outcomes and late effects of transplant. As survival from transplant has improved, more attention is now given to long-term disease outcomes and quality of life. Therefore, the goal of conditioning is to give the least toxic regimen with minimal short- and long-term side effects but still achieve cure of the underlying condition. This review will focus on newer conditioning regimens, how they have changed, and possible future directions. It is important to note that success does not simply depend on which conditioning chemotherapeutic agents are employed but on a combination of factors such as additional serotherapy, timing and dosage, and stem cell source. In almost all cases, preparative conditioning with a combination of chemotherapeutic agents, with or without monoclonal antibodies, is required for successful engraftment and stable robust long-term immune reconstitution.

## Definition

**Fig. 1:**
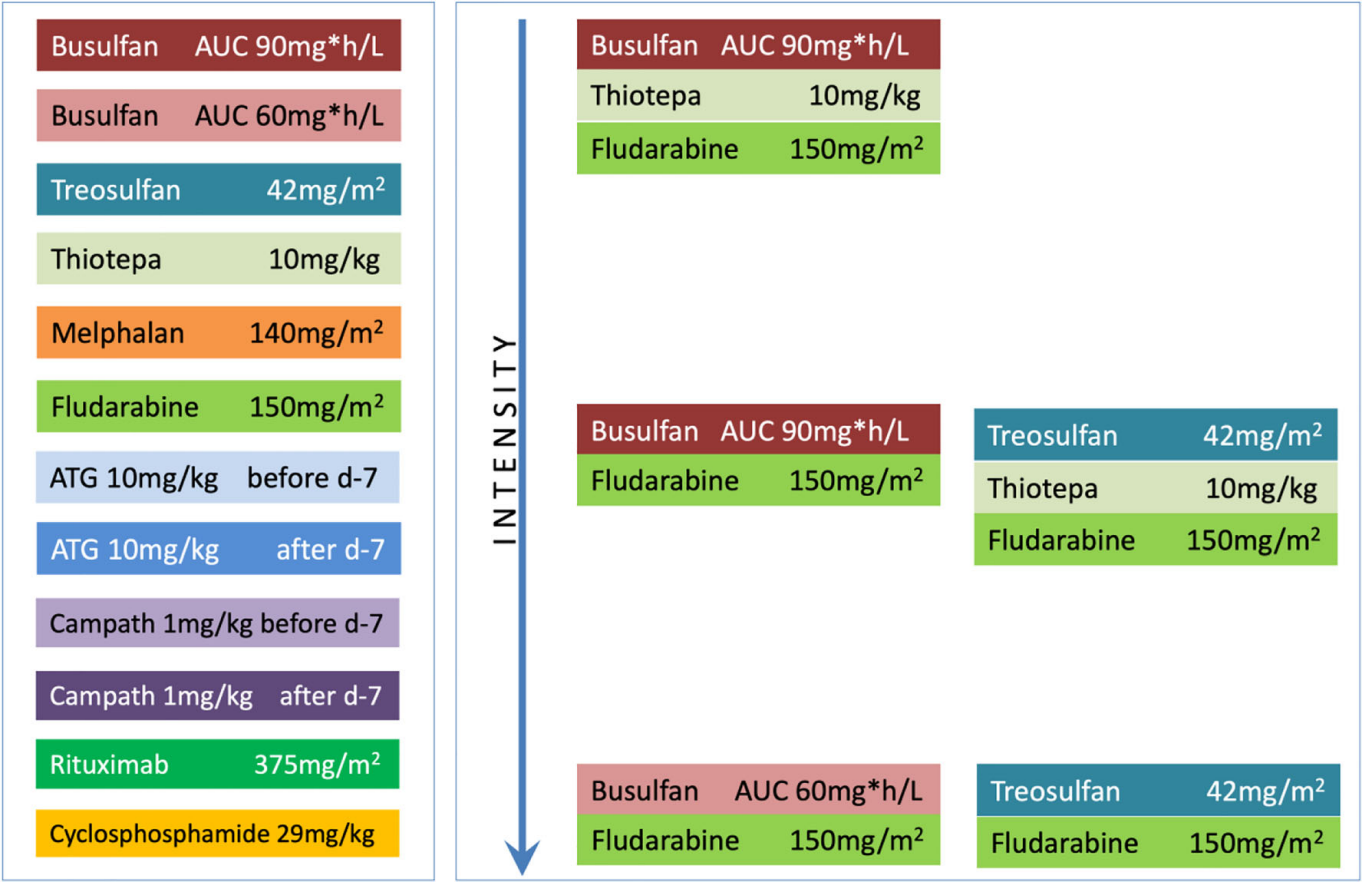
Intensity of conditioning regimen according to chemotherapy, pharmacokinetic guided dosing, timing of serotherapy, and combination of chemotherapy

The intensity of the conditioning regimen can vary substantially and has been classified as myeloablative conditioning (MAC), reduced toxicity conditioning (RTC), reduced intensity conditioning (RIC), and minimal intensity conditioning (MIC) in decreasing order (Fig. 1). MAC, consisting of alkylating agents with or without total body irradiation (TBI), is expected to myeloablate the recipient’s hematopoiesis which does not allow for autologous hematological recovery. This aims to prevent rejection by the use of supralethal chemotherapy to remove host-versus-graft reaction and create marrow niche space for donor stem cells. Newer myeloablative chemotherapy agents are being explored to reduce toxicity and enable safer HCT. These reduced toxicity conditioning (RTC) regimens, including pharmacokinetic targeted busulfan-fludarabine (Bu-Flu) and treosulfan-fludarabine, have a comparable myeloablative effect with conventional MAC but reduced organ toxicities. Compared to MAC, RIC has been traditionally characterized by reversible myelosuppression in the absence of stem cell rescue, reduced regimen-related toxicity, and a higher incidence of mixed chimerism. MIC is strictly non-myeloablative, does not eradicate host hematopoiesis, and allows relatively rapid autologous hematopoietic recovery without a transplant, but adequately myelosuppresses the recipient to enable at least partial donor engraftment.

## Myeloablative Conditioning Regimens in PID

Historically, conditioning therapy prior to HCT in PID was based on the combination of alkylators busulfan and cyclophosphamide. However, many children with PID have significant comorbidities at the time of HCT, and these conventional myeloablative preparative regimens are associated with significant toxicity and a relatively high incidence of transplant mortality, as well as long-term sequelae. While initial results may have been acceptable, appreciation of acute conditioning toxicities and recognition of long-term sequelae mean that few centers now approach transplantation of PID patients with conventional myeloablative preparative regimens (Table 1) [6–9].

**Table 1:** Outcome of HCT in PID after myeloablative conditioning regimens

Author, Year	Year of HCT	No. of patients/diagnosis	Median age at HCT (range), years	Donor and stem cell source	Conditioning regimen	OS
Fisher, 1994 [6]	1977–1991	149 non-SCID PID received 171 transplants	Range 0.1–16	65 MSD/MFD 6 MUD 78 MMUD	Bu+Cy 12 additional TBI	Before 1985, 51.7% After 1985, 81.5%
Klein, 1995 [7]	1981–1993	19 MHC class II deficiency (7 s HCT)	1.4 (0.5–9.5)	8 MFD marrow 1 MMFD marrow 10 HID marrow All 7 s HCT used HID	*MFD* Bu20mg/kg + Cy 200 mg/kg or Cy 50 mg/kg + ALG or Cy 50 mg/kg + CCNU 300 mg/m^2^ + procarbazine 280 mg/kg + ALG *MMFD* Bu 16 mg/kg + Cy 200 mg/kg or Bu 20 mg/kg + Cy 200 mg/kg + anti-LFA-1 antibody or Bu 20 mg/kg + Cy 200 mg/kg + anti-LFA-1 antibody + anti-CD2 antibody	47%
Antoine, 2003 [8]	1968–1999	1082 HCT in 919 PID patients 566 HCT in 475 SCID patients 512 HCT in 444 non-SCID PID patients	SCID: 5.5 months Non-SCID: 34.6 months	88% marrow 12% PBSC 0.7% CB T cell depletion: 91% MD 41% UD marrow	205 SCID: unconditioned 361 SCID: Bu 8 mg/kg + Cy 200 mg/kg 512 non-SCID; Bu 16 mg/kg + cy 200 mg/kg	SCID: 77% MD vs 54% in MMD Non-SCID: 71% MFD vs 42% MUD vs 59% MMD
Renella, 2006 [9]	1981–2004	15 MHC class II deficiency	1.5 (0.3–5.4)	13 MFD marrow 2 MUD marrow	Bu 16-20 mg/kg + Cy 200 mg/kg + ATG in MUD	53%

*ALG* antilymphocyte globulin, *Bu* busulfan, *CB* cord blood, *CCNU* lomustine, *Cy* cyclophosphamide, *HID* haploidentical donor, *MD* matched donor, *MFD* matched family donor, *MMD* mismatched donor, *MSD* matched sibling donor, *MMUD* mismatched unrelated donor, *MUD* matched unrelated donor, *OS* overall survival, *PID* primary immunodeficiency, *SCID* severe combined immunodeficiency, *TBI* total body irradiation, *UD* unrelated donor, *WAS* Wiskott-Aldrich syndrome

## RTC Regimens in PID

The use of reduced toxicity conditioning regimens are now generally preferred for patients with PID as there is no malignant disease to eradicate, stable mixed chimerism achieves cure for many diseases, and many patients enter HCT with chronic infections and end-organ comorbidities. Additionally, many patients are infants at the time of transplant and may be more susceptible to toxicity [10]. Less toxic regimens may reduce early and late adverse effects, particularly infertility [4•]. There are several reduced toxicity regimens that have been utilized by investigators in PID (Table 2) [14•, 49, 50].

**Table 2:** Outcome of HCT in PID according to reduced toxicity conditioning regimens

Author, year	Year of HCT	No of patients/diagnosis	Median age at HCT (range), years	Donor and stem cell source	Conditioning regimen and GvHD prophylaxis	Median day of N engraftment	VOD, *n*	aGvHD %	cGvHD %	OS %	ES %	Graft failure %	Second procedure, *n*	Latest donor chimerism/remarks
Fludarabine and treosulfan														
Slatter, 2018 [11]	2006–2013	160 39 SCID 20 WAS 17 CGD 18 HLH 66 Other PID:	1.36 (0.1–18.3)	29 MSD/MFD 73 MUD 54MMUD 4 HID 49 marrow 70 PBSC 41 CB	Flu 150 mg/m^2^ + Treo 42 g/m^2^ (36g/m^2^ if < 1 year; 30 g/m^2^ for SCID) + alemtuzumab 0.3 to 1.0 mg/kg GvHD prophylaxis: CSA/MMF	NA	0	I–IV: 46 III–IV: 9	15	2-year OS: 88 5-year OS: 78	2-year ES: 88 5-year ES: 78	3	4 s HCT for graft loss or poor immune reconstitution 5 unconditioned boost 3 DLI	PBSC was associated with better donor myeloid chimerism without an increased risk of GvHD
Morillo-Gutierrez, 2016 [12]	2006–2015	70 CGD	8.9 (IQR 3.8–19.3)	13 MSD/MFD 44MUD 12 MMUD 1 HID 36 marrow 32 PBSC 1 TCR ∝β/CD19 depleted PBSC 1 CB	46 Flu150mg/m^2^ + Treo 42 g/m^2^ (36g/m^2^ if < 1 year) Alemtuzumab (*n* = 39) or ATG (*n* = 18) or no serotherapy (*n* = 13) 15 Flu + Treo + TT + alemtuzumab or ATG 9 other Treo-based conditioning regimen GvHD prophylaxis: CSA ± MMF or MTX	17 (IQR 15–35)	0	I–II: 39 III–IV: 12	13	91.4	81.4	12	8 (2 unconditioned boost; 3 DLI; 5 conditioned 2nd HCT [2 had DLI])	Myeloid ≥ 95%: 80% surviving patients
Slatter, 2015 [13]	2005–2010	316 144 PID 39 IMD 70 H-globinopathy 32 histiocytic disorders 24 marrow failure 2 autoimmune disease 5 others	< 1 year, *n* = 95 1–12 years, *n* = 189 > 12 years, *n* = 32	94 MSD/MFD 29 MMRD 39 MUD 16 MMUD 138 undefined UD 167 marrow 8 marrow + CB 3 marrow + PBSC 87 PBSC 1 PBSC + CB 50 CB	106 Flu 150 mg/m^2^ + Treo 42 g/m^2^ 98 Cy 200 mg/kg + Treo 42 g/m^2^ 104 Flu 150 mg/m^2^ + Treo 42 g/m^2^ + TT 8 mg/kg 8 Flu 150 mg/m^2^ + Treo 42 g/m^2^ + melphalan GvHD prophylaxis: 284 CSA alone 100 CSA + MMF 101 CSA + MTX	NA	0	I–IV: 38 III–IV: 10	NA	83	76	5.1	NA	NA
Burroughs, 2014 [14]	2009–2013	31 6 IPEX 5 CGD 2 other PID 6 HLH 6 BM failures 6 RBC disorders	10.7 (0.4–30.5)	4 MSD 27 MUD 29 marrow 2 PBSC	Flu 150 mg/m^2^ + Treo 42 g/m^2^ Serotherapy: 22 ATG GvHD prophylaxis: Tacrolimus + MTX	21 (range, 12–46)	0	II–IV: 62 III–IV: 10	21	90	NA	3	2 s HCT	*ATG patients:* 19 (86%) full or high level of mixed CD3 chimerism 3 (14%) low-level mixed donor CD3 chimerism *No ATG patients:* 6 full/high level of mixed CD3 chimerism 2 low-level mixed donor CD3 chimerism 1 graft failure
Dinur-Schejter, 2015 [15]	2009–2013	45 HCT in 44 patients 12 SCID 5 severe congenital neutropenia 2 WAS 2 CGD 1 HLH 10 PID 5 thalassaemia 5 osteopetrosis 3 IMD 4 others	1.5 (0.1–15.1)	19 MSD/MFD 3 MMFD 14 MUD 9 unrelated CB	19 Flu + Treo 6 Cy + Treo 20 Flu + Treo + TT (Treo 36 g/m^2^ for < 1 year; 42 g/m^2^ for > 1 year) Serotherapy: 9 no serotherapy 26 ATG 8 Alemtuzumab 1 OKT3	Flu/Treo/TT: 18.4 Flu/Treo: 25.3 Cy/Treo: 19.5	1	I–IV: 44.4 III–IV: 27	18.9	71	55	14	3 s HCT (one had a further 3rd HCT)	Full: 31 (72%) Mixed: 6 (28%)
Lehmberg, 2014 [16]	2010–2012	19 HLH	3.9 (0.2–22)	1 MRD 6 MUD 9 MMUD HID 1 17 marrow 1 PBSC 1 CD34 selected PBSC for HID	16 Flu150mg/m^2^ (3 Flu 160-180 mg/m^2^) + Treo 42 g/m^2^ (36g/m^2^ if < 12 kg) Alemtuzumab 0.3 mg—1.0 mg/kg 14 additional TT 10 mg/kg (7 mg/kg if < 12 kg) GvHD prophylaxis: 2 CSA alone 7 CSA + MMF 9 CSA + MTX 1 Tacrolimus + MMF	20 (range 11–62)	1	I–II: 21 III–IV: 1 patient after DLI	No	100	NA	11 (*n* = 2)	2 s HCT (1 1° graft failure after HID; 1 2° graft failure) 6 DLI	WB > 95%: 10 WB 75–95%: 2 WB 20–74%: 4
Beier, 2013 [17]	2003–2009	53 non-malignant patients 10 SCID 4 CGD 2 HLH 2 WAS 11 other PID 3 osteopetrosis 9 H-globinopathy 9 BM failure 1 IMD 2 0thers	4.8 (0.1–20.1)	16 MSD/MFD 1 MMFD 1 MUD 25 MUD I HID 2 CB + HID 36 marrow 11 PBSC 1 CB 2 CB + PBSC 2 NA	15 Flu + Treo (1 additional radioimmunotherapy) 32 Flu + Treo + TT 5 Flu + Treo + melphalan Serotherapy 4 None 19 ATG 3 ATG + OKT3 1 ATG + alemtuzumab 16 alemtuzumab 1 alemtuzumab + rituximab 1 rituximab 5 OKT3	20	0	I–IV: 32 III–IV: 4	6 (*n* = 3)	87	NA	4	NA	Full: 46 (87%)
Slatter, 2011 [18]	2006–2009	70 26 SCID 7 Omenn syndrome 7 WAS 4 HLH 4 LAD 4 CGD 2 IPX 16 other PID	0.7 (0.1–14.6)	21 MSD/MFD 45 MUS 4 HID	40 Flu150mg/m^2^ + Treo 42 g/m^2^ 30 Flu150mg/m^2^ + Cy 200 mg/kg 53 alemtuzumab 0.3 to 1.0 mg/kg	NA	2 in Cy group	I–IV: 26 III–IV: 10	6	81 Flu: 85% Cy: 77%	NA	3 (*n* = 2_	1 had both top-up and second conditioned HCT	57% full donor chimerism 43% stable mixed chimerism
Busulfan ± fludarabine														
Dvorak, 2019 [19]	2011–2017	10 4 typical SCID 6 leaky SCID	5 mos (range, 2–108 mos)	2 MUD 2MMUD 6 HID Marrow for MUD/MMUD CD34 selected PBSC or HID	Bu with target AUC 30 mg*hr./L ATG or alemtuzumab For patients with any T cells: Additional Flu 160 mg/m^2^ For patients with NK cells: Additional TT 10 mg/kg 2 had plerixafor 9 h prior to each dose of Bu	16 (range, 14–23)	0	II–IV: 2 patients	0	100	NA	10	1 additional HCT	Median myeloid at one-year post HCT 14% (range, 2–100%) 6 had full T- and B cell reconstitution 3 had no B cell recovery (2 had rituximab for autoimmunity post-HCT) 3 had B cell autoimmunity
Güngör, 2015 [20]	2003–2015	56 CGD	12.7 (IQR 6.8–17.3)	21 MSD/MFD 25 MUD 10MMUD 45 marrow 11 PBSC	Flu 150 mg/m^2^ Bu with target AUC 45–65 mg*hr./Lxh Serotherapy ATG for MFD Alemtuzumab for MUD	19 (IQR 16–22)	0	III–IV: 4	7	93	89	5	3 s HCT	Myeloid > 90%: 52 (93%)
Jacobsohn, 2004 [21]	2000–2004	13 6 PID 4 H-globionopathy 3 IMD	5.2 (IQR, 0.6–11.1)	4 MSD 1MMFD 6 MUD 2 unrelated CB 11 PBSC	Flu 150 mg/m^2^ Bu with target AUC 3800 to 4200umol x min ATG GvHD prophylaxis CSA ± MMF	18 (IQR, 14–25)	0	II–IV: 8	25	84	NA	15 (2 h-globinopathy)	none	72% full donor chimerism
Fludarabine and melphalan														
Allen, 2018 [22]	2013–2015	34 HLH 12 PID	2.3 (0.4–28)	7 MSD 1 MMRD 25 MUD 13 MMUD All had marrow	Flu 150 mg/m^2^ Melp 140 mg/m^2^ Alemtuzumab 1 mg/kg GvHD prophylaxis CSA and steroid	13	0	II–IV: 17.4 III–IV: 10.9	26.7	18-month OS: 66.9%	60.9% with second procedure 39.1% without intervention	Primary: 4 Secondary: 4	2 s HCT	57% had full chimerism in all cell lines 42% had stable mixed chimerism
Fox, 2018 [23]	2004–2014	29 PID	24 [11, 12, 16, 18–48]	11 MFD 13 MUD 5 MMUD	Non-CGD Flu 150 mg/m^2^ Melp 140 mg/m^2^ Alemtuzumab 100 mg CGD Flu 150 mg/m^2^ Meph 10 mg/m^2^ or Bu 9.6 mg/kg Alemtuzumab or ATG GvHD prophylaxis CSA	13 (IQR, 11–17)	0	I–II: 45 III–IV: 3	Limited: 34 Extensive: 1	1-yr: 85.2	1-year: 85.7	None	None	85% full chimerism
Marsh, 2010 [30]	2003–2009	40 HLH	1 (0.1–16)	7 MFD 33 MUD 36 marrow 2 PBSC 2 CB	26 RIC Flu 150 mg/m^2^ (5mg/kg if < 10 kg) Melp 140 mg/m^2^ (4.7 mg/kg if < 10 kg) Alemtuzumab 14 MAC Bu 14 mg/kg Cy 200 mg/kg 12 additional etoposide 30 mg/kg GvHD prophylaxis CSA or tacrolimus + steroid/MTX	MAC: 14.5 RIC: 10	NA	II-III MAC: 14 RIC: 8 (*p* = 0.317)	MAC: 0 RIC: 12% limited	MAC: 43% RIC 89% (*p* = 0.0036)	NA	None	3 CD34+ boost 14 DLI	MAC: 18% mixed RIC: 65% mixed Mixed chimerism in RIC was less in patients who received distal aleumtuzumab (29%) vs 79% in proximal alemtuzumab (*p* = 0.02)
Rao, 2005 [49]	1998–2001	33 6 SCID 27 non-SCID	5.9 (0.19–18)	22 MUD 11 MMUD All marrow	Flu 150 mg/m^2^ Melp 140 mg/m^2^ Alemtuzumab 1 mg/kg CSA	13 (range, 8–34)	0	II–IV: 9	Limited: 0 Extensive: 3	94%	NA	NA	NA	55% had full chimerism 32% had high level mixed chimerism 6.5% had low level mixed chimerism 6.5% very low mixed chimerism
Amrolia, 2000 [31]	NA	8 3 SCID 1 XLP/HLH 2 CID 2 CD40 ligand def	6.5 (range, 0.75–18)	2 MSD 6 MUD All marrow	Flu 150 mg/m^2^ Melp 140 mg/m^2^ ALG 10 mg/kg GvHD prophylaxis CSA and steroid	13 (range, 9–17)	0	I: 50 II–IV: 0	limited cGvHD, *n* = 1	88	NA	1 patient	None	4 had 100% donor chimerism 3 had mixed chimerism
Fludarabine and low-dose TBI														
Burroughs, 2010 [36]	NA	2 IPEX	0.75, 16	2 MUD 1 marrow 1 PBSC	Flu 90 mg/m^2^ TBI 4Gy GvHD prophylaxis CSA and MMF	16, 17	0	2 had grade II	1 severe	Both alive	Both engrafted	None	None	Full immune function and normal FOXP3 protein expression
Burroughs, 2007 [35]	1998–2006	14 PID	Range 0.5–30	8 MFD 8 MUD 8 marrow 5 PBSC 1 CB	Flu 90 mg/m^2^ (*n* = 13) TBI 2Gy (*n* = 14) GvHD prophylaxis CSA and MMF	15 (range 5–23)	0	II: 71 III–IV: 7	Extensive: 47	62	62	1	1 unconditioned PBSC for slipping myeloid chimerism 1 conditioned HCT for persistent thrombocytopenia 1 DLI for low donor CD4 and CD8 chimerism 1 conditioned HCT for graft failure	5 mixed chimerism 8 full donor chimerism
Antibody-based conditioning														
Schulz, 2011 [44]	2003–2007	14 non-malignant 4 SCID 2 CGD 2 Hyper IgM 2 other PID 4 H-globinopahty	7.5 (range, 1–20)	3 MFD 1 MMFD 8 MUD 2 HID 8 marrow 4 PBSC 2 TCD-PBSC	^90^Y-labeled anti-CD66 antibody at Day −14 Fludarabine 160 mg/m^2^ Melphalan 70-140 mg/m^2^ ATG for mismatched donor and unrelated donor	NA	0	II: 36 III–IV: 0	limited, *n* = 2 extended, *n* = 3	88	81	*n* = 1	1	9 had 100% chimerism 2 had mixed chimerism
Straathof, 2009 [24]	1999–2002	16 8 SCID 1 MHC class II def. 1 IPEX 1 HLH 1 DKC + SCID 1 Ligase 4 def. 1 CD40 ligand def. 2 Other PIDs	0.7 (range, 0.4 to 11.4)	5 MSD 9 MUD 2 MMUDD 40 marrow 12 PBSC 1 PBSC + marrow 17 CB	Anti-CD45 1.6 mg/kg (day − 5 to − 2) Flu 150 mg/m^2^ Alemtuzumab 0.3 to 0.6 mg/kg GvHD prophylaxis CSA and MMF	9.5 (range 1–15)	0	II–IV: 38 III–IV: 19	31	81	95	3	1 s HCT	Median myeloid: 100% (range, 41–100%) Median lymphocyte: 100% (range, 54–100%)

*1°* primary, *2°* secondary, *aGvHD* acute graft-versus-host disease, *ALG* antilymphocyte globulin, *ATG* anti-thymocyte globulin, *AUC* area under curve, *BM* bone marrow, *BU* busulfan, *CB* cord blood, *CGD* chronic granulomatous disease, *cGvHD* chronic graft-versus-host disease, *CSA* ciclosporin, *def* deficiency, *DLI* donor lymphocyte infusion, *ES* engrafted survival, *Flu* fludarabine, *H-globinopathy* hemoglobinopathy, *HID* haploidentical donor, *HLH* hemophagocytic lymphohistiocytosis, *IMD* inherited metabolic disease, *IQR* interquartile range, *MMF* mycophenolate mofetil, *MMRD* mismatched related donor, *MMUD* mismatched unrelated donor, *MSD* matched sibling donor, *MUD* matched unrelated donor, *MTX* methotrexate, *N* neutrophil, *NA* not available, *OS* overall survival, *PID* primary immunodeficiency diseases, *SCID* severe combined immunodeficiencies, *Treo* treosulfan, *TT* thiotepa, *vs* versus, *WAS* Wiskott-Aldrich syndrome, *WB* whole blood

### Fludarabine and Treosulfan

Treosulfan (L-treitol-1,4-bis-methanesulfonate) is a prodrug and a water-soluble bifunctional alkylating agent which has been used for many years as treatment for various neoplasms, but more recently as part of conditioning for HSCT. In addition to myeloablative properties, it has marked immunosuppressive properties which contribute to the achievement of stable engraftment posttransplant. It causes relatively low organ toxicity compared to high-dose busulfan and cyclophosphamide leading to fewer complications such as veno-occlusive disease of the liver.

The first successful allogeneic transplant in a child using treosulfan was performed in 2000 and since then many reports have confirmed its efficacy and safety in both malignant and non-malignant disorders [11••, 12•, 13, 14•, 15–18]. Slatter et al. first published results of 70 children with PID who received treosulfan in combination with either cyclophosphamide (*n* = 30) or fludarabine (*n* = 40) with an overall survival of 81% (median follow-up 19 months) equivalent in those aged less or greater than 1 year at time of transplant [13]. Toxicity was low but worse after cyclophosphamide, and T cell chimerism was significantly better after fludarabine [18]. Slatter et al. more recently reported 160 patients who had received conditioning with treosulfan and fludarabine achieving a probability of 2-year survival of 87.1% with a high level of complete or stable mixed chimerism in the diseased cell lineage, sufficient to cure disease [11••]. There was a high survival rate in children transplanted under 1 year of age in whom toxicity can be a problem with conventional and other reduced intensity conditioning regimens [24, 25]. A 100-day survival of 94% demonstrated the low toxicity of this regimen making it suitable for patients with PID who often have infection and organ damage prior to HCT. In this series, a higher level of myeloid chimerism was found in recipients of PBSC compared to CB and BM, without an increased risk of grade III/IV acute or chronic graft-versus-host disease (GvHD). This highlights the importance of the whole transplant package including stem cell source and serotherapy when tailoring therapy [26].

Excellent results were reported by Lehmberg et al. in 19 patients with hemophagocytic lymphohistiocytosis (HLH) following HCT with treosulfan, fludarabine, alemtuzumab, with or without thiotepa, all of whom survived with a median follow-up of 16 months [16].

Haskologlu et al. reported 15 patients with PID who had a high risk of developing transplant-related toxicity due to previous lung and liver damages and were given treosulfan-based conditioning [27]. At 32 months follow-up, the overall survival was 86.7% with excellent chimerism and low conditioning associated morbidity despite the high-risk population.

Mixed chimerism is sufficient to achieve cure in some non-malignant disorders, but the specific diagnosis and level of chimerism needed to achieve cure must be taken into account when balancing the need for increased myeloablation against short- and long-term toxicities from the conditioning regimen. The addition of thiotepa is common in order to increase the intensity of the regimen, but there are few reports of any comparison in outcomes comparing treosulfan and fludarabine with or without additional thiotepa. Yael Dinur-Schejter et al. reported 44 patients with non-malignant diseases: 19 received treosulfan with fludarabine 66.7% of whom achieved complete engraftment compared to 94.7% of 20 patients who received additional thiotepa, but this did not translate into any significant difference in overall or event free survival [15].

### Fludarabine and Busulfan

Traditionally, busulfan (Bu) was used in combination with cyclophosphamide (Cy) as the standard myeloablative conditioning regimen for HCT for both malignant and non-malignant disorders in both adult and pediatric patients. Cyclophosphamide is increasingly being substituted with fludarabine (Flu), a nucleoside analogue with immunosuppressive properties, to provide a less toxic but equally effective regimen [19, 21, 28].

Harris et al. compared 1400 children who received Bu-Cy to 381 who received Bu-Flu. Busulfan doses were comparable between the 2 groups and the majority had pharmacokinetic monitoring. Eight hundred and three had non-malignant disorders including 195 with PID who received Bu-Cy and 86 who received Bu-Flu. Nine hundred and seventy-eight had malignant disorders. Children receiving Bu-Flu for non-malignant conditions experienced less toxicity than those receiving Bu-Cy, but survival was comparable. Children with malignancy had shorter postrelapse survival with Bu-Flu than Bu-Cy although transplant-related mortality and relapse were similar [29].

The pharmacokinetics of busulfan have been studied extensively and the use of a lower target area under the curve (45–65 mg/L × h) combined with fludarabine has been pioneered by Tayfun Güngör and colleagues in Zurich. Particularly impressive results have been seen using this regimen for patients with chronic granulomatous disease (CGD). Fifty-six children and young adults with CGD were reported, many of whom had high-risk features such as intractable infections and autoinflammation. Twenty-one HLA-matched related-donor and 35 HLA-matched unrelated-donor transplants were done. The 2-year probability of overall survival was 96% (95% CI 86∙46–99∙09), and of EFS was 91% (79∙78–96∙17). Graft-failure occurred in 5% (three of 56) of patients. The cumulative incidence of acute GvHD of grade III–IV was 4% (two of 56) and of chronic GvHD was 7% (four of 56). Stable (≥ 90%) myeloid donor chimerism was documented in 52 (93%) surviving patients [20••].

Dvorak et al. have recently reported the result of the use busulfan at a lower target area under the curve (30 mg/L × h) alone or in combination with fludarabine or thiotepa in 10 patients with severe combined immunodeficiency. All the patients survived, one patient required second HCT, and 3 had no B cell reconstitution [19].

## RIC in PID

### Fludarabine and Melphalan

Increasing recognition of the significant toxicities associated with conventional doses of busulfan and cyclophosphamide, particularly in very young infants and especially in those with pre-existing end organ damage, led to the adoption of immunosuppressive-based, rather than myelo-ablative-based regimens, with fludarabine and melphalan. The results, principally in those with significant preexisting comorbidities, were striking with significantly improved early survival [22, 23, 30, 31, 49]. However, donor chimerism was not always optimal, and there was a high incidence of late viral reactivation, and late onset acute GvHD. Furthermore, toxicities in infants < 1 year of age remained significant [25]. Melphalan in particular has been associated with cardiac toxicities [32]. Good results have been reported for patients with hemophagocytic lymphohistiocytosis [33]. Patients with X-linked inhibitor of apoptosis protein (XIAP) deficiency, which is difficult to transplant, also have good outcomes reported using fludarabine and melphalan-based regimens [34]. It has been used in adults with PID with good transplant survival [23]

While the approach remains attractive in terms of reduced toxicities, concerns regarding late graft failure and high mortality in the < 12-month-aged infants remain.

## Minimal Intensity Conditioning for PID

### Fludarabine and Low-Dose TBI

Burroughs et al. from the Seattle group have reported the transplant outcome of using fludarabine and low-dose TBI in 14 PID patients with significant preexisting organ dysfunction and infections. All received posttransplant GvHD prophylaxis with cyclosporin and mycophenolate mofetil but no serotherapy. Overall survival at 3 years was 62%, but there were high rates of acute (79%) and extensive chronic GvHD (47%) [35]. One had graft failure and an additional three patients required a second procedure for decreasing chimerism. Of 10 evaluable patients, 8 had correction of immune deficiency with stable chimerism. However, the high rate of GvHD has limited the broader use of this conditioning regimen in children with PID [35, 36].

### Antibody-Based

While conditioning regimens have undoubtedly become less toxic, the ability to achieve donor chimerism without the use of chemotherapeutic agents, particularly in patients with non-malignant disease, is extremely attractive. Furthermore, some primary immunodeficiencies have significant toxicities associated with the administration of alkylating agents, due to the nature of the molecular defect, leading to serious long-term effects or early mortality [37–39]. A number of different strategies have been employed to minimize the exposure to chemotherapeutic agents by the use of antibodies to aid stem cell engraftment, with or without adjunct chemotherapy.

### Anti-CD45 Antibodies

CD45 is selectively expressed on all leucocytes and hematopoietic progenitors but is absent on non-hematopoietic tissues. Straathoff and colleagues studied 16 patients with PID who were less than 1 year of age or had significant preexisting comorbidities and were felt not suitable for conventional reduced intensity conditioning [24]. The conditioning regimen was comprised of alemtuzumab 0.2 mg/kg daily for 3 days for unrelated donors, or 0.1 mg/kg daily for 3 days for matched sibling donors on day − 8 to day − 6, clinical grade rat anti-CD45 (YTH24·5and54·12) 0.4 mg/kg on day − 5 to day − 2, fludarabine (30 mg/m^2^ daily for 5 days on day − 8 to day − 4) and cyclophosphamide (300 mg/m^2^ daily for 4 days on day − 7 to day − 4). Twelve patients were alive and well at the end of the study, one failed to engraft and was successfully re-transplanted, and 3 died—none of conditioning toxicity. Donor chimerism was variable but high level and sufficient to cure disease in the survivors.

### Radioimmunotherapy

Radioimmunotherapy is an attractive concept for conditioning of patients with PIDs as it exploits of the physical cytotoxic effect of radiation and reduces the toxicity to other organ systems by its internal application and the conjugation of radioisotopes to specific antibodies [40]. Radioisotopes emitting α, β or γ-radiation of calculated intensity can be brought in direct proximity to the cells of interest. This enables malignant cells to be eradicated or benign hematopoietic cells to be depleted as part of conditioning before autologous or allogeneic HSCT. The method was developed to allow better and more specific control of malignant cells in the setting of HSCT without an increase in non-relapse mortality. Considerable clinical data was accumulated with conjugates of ^90^Yttrium or ^131^Iodine to anti-CD20 antibodies in the treatment of patients with refractory or recurrent B cell non-Hodgkin lymphoma (B-NHL). The drugs were used in combination with chemotherapy to prepare patients for autologous and allogeneic stem cell transplantation. This experience resulted in the approval of two drugs (Zevalin® and Bexxar®) by the FDA at the beginning of the century [40].

The use of RIT for the treatment of leukemias or for myeloablation in non-malignant disease until present is limited to clinical studies. A conjugate of ^131^Iodine to anti-CD45-antibody was explored in the treatment of patients with AML and high-risk MDS, again a combination of RIT with conventional myeloablative or immunosuppressive drugs was used for conditioning before allogeneic HSCT [41, 42]. CD45 is expressed on most AML and ALL blasts as well as on virtually all developing and mature cells of normal hematopoiesis. Radiolabeled anti-CD45 antibody doses up to 43 Gy were administered to the bone marrow in combination with RIC and allogeneic transplantation with good tolerance and without additional toxicity in younger adult patients with AML and MDS [43]. For children, limited published data exists for the use of RIT for pretransplant conditioning. A conjugate of ^90^Yttrium to an antibody targeting CD66 was used in combination with melphalan and fludarabine or TBI for the treatment of children with considerable comorbidities with malignant and non-malignant disease. ^90^Yttrium emits pure β-radiation with a maximum range of 11 mm and a half-life of 2.7 days [44]. With these qualities, no isolation of the pediatric patients was necessary, but the dosimetry had to be performed with another isotope, emitting γ-radiation to be detected in a γ-camera. CD66 is abundantly present on mature myeloid cells but usually not expressed on malignant blasts. The therapeutic principle of RIT with this antibody in malignant disease therefore relies on the so-called cross-fire effect, which describes the indirect depletion of blasts by binding of the antibody to cells in close proximity [40]. In order to avoid graft rejection in unrelated or mismatched grafts, recipients received serotherapy with ATG in this setting. Fifteen of 16 children with non-malignant disease survived the procedure, 13/15 with complete donor chimerism. The Kaplan-Meier estimation for disease-free survival at 24 months was 94%. This clearly documented feasibility of and reliable myeloablation by RIT in children and young adults with non-malignant disease.

### Anti-CD117 Antibodies

The molecule CD117 (c-Kit receptor) is expressed on hematopoietic stem cells at all stages of development. Interactions with the ligand of CD117, stem cell factor, are crucial for hematopoietic stem cell survival, and this signaling pathway plays a critical role in the homing, adhesion, maintenance, and survival of hematopoietic stem cells in the hematopoietic niche. Preclinical studies demonstrated that using an antibody against CD117 to impede CD117-stem cell factor signaling selectively depleted hematopoietic stem cells with no effect on differentiated progenitor or mature cell lineages, and enabled engraftment of donor cells [45]. A clinical trial is currently in progress using anti-CD117 antibody alone to treat patients with primary immunodeficiencies (AMG191 Conditioning/CD34 + CD90 Stem Cell Transplant Study for SCID Patients, ClinicalTrials.gov Identifier: NCT02963064). The early results of this dose finding study show that some donor stem cell chimerism, leading to donor T and B lymphocyte chimerism can be achieved [46]. These preliminary data are extremely exciting and potentially lead the way to a step change in approaches to conditioning in patients with PIDs.

## Conditioning for Haploidentical Donor Transplant

As the outcomes of HCT using newer T cell depletion methods have improved, there is an increasing number of haploidentical transplants performed for both SCID and non-SCID PID. Various non-myeloablative conditioning regimens have been used in T-deplete and T-replete haploidentical transplant (Table 3) [5••, 47, 48, 51]. The Great North Children’s Hospital (GNCH) group in Newcastle has used fludarabine, treosulfan, ATG (Grafalon), and ritixumab for patients who received CD3 TCR ab/CD19 depleted peripheral blood stem cells. Patients with non-SCID PID received additional thiotepa. The overall survival was comparable with family and unrelated donor transplant using a similar conditioning regimen [18, 51]. Neven et al. reported the outcome of Bu-Flu in 22 patients with PID received haploidentical transplant using posttransplant cyclophosphamide. The overall survival and donor chimerism were good, but 48% had acute GvHD and 24.2% had chronic GvHD.

**Table 3:** Outcome of haploidentical donor transplant in PID using modern T lymphocyte depletion strategies and various conditioning regimens

Author, year	Year of HCT	No of patients/diagnosis	Median age at HCT (range), years	Donor and stem cell source	Conditioning regimen and GvHD prophylaxis	Median day of N engraftment	VOD %	aGvHD %	cGvHD %	OS %	ES %	Graft failure %	Second procedure, *n*	Latest donor chimerism/remarks
Fludarabine and treosulfan														
Neven, 2019 [48]	2014–2017	22 PID 5 osteopetrosis 21 first HCT 6 s HCT	1.5 (0.2–17)	27 HID All marrow	20 MAC with Bu-pk + Flu 160 mg/m^2^ (4 received additional Cy 28 mg/kg) Serotherapy: rituximab plus alemtuzumab/ATG 7 had RIC (1 first HCT and 6 s HCT) GVHD prophylaxis CSA MMF PTCy 50 mg/kg on day 3 + 4	19 [11–13, 15–34]	11	II–IV: 48 II: *n* = 10 III: *n* = 2	24.2	77.7	77.7	*n* = 2	1	24 full chimerism 1 mixed chimerism
Shah, 2018 [5]	2012–2016	25 PID 3 for refractory GvHD	1.75 (0.28–10.3)	23 HID 2 MMUD TCR ab/CD 19 depleted PBSC	Flu 150 mg/m^2^ Treo 36-42 mg/m^2^ TT 10 mg/kg 24 had serotherapy (ATG/alemtuzumab) 6 had rituximab 3 SCID: unconditioned GvHD prophylaxis: CSF/MMF	25 [10–19, 21, 24–28, 49, 50]	0	II–IV: 22	None	83.9	80.4	*n* = 1	1	76.1% full donor chimerism 5 had high T cell but mixed myeloid chimerism (2 unconditioned)
Rastogi, 2017 [47]	2013–2016	8 PID	4.9 (0.8–12)	7 HID 1 MUD Unmanipulated marrow/PBSC	5 Flu 160 mg/m^2^ + Cy 29 mg/kg + TBI 2Gy (3 had additional TT) + ATG/alemtuzumab 2 Flu 160 mg/m^2^ + Treo 42 mg/^2^ 1 Flu 160 mg/m^2^ + Bu 3.2 mg/kg GVHD prophylaxis Tacrolimus MMF PTCy 50 mg/kg on Day 3 + 4	Mean 17	NA	I–II: 3 patients II–IV: none	2 limited	75	75	None	None	All full donor chimerism
Balashov, 2015 [51]	2012–2014	37 PID 5 SICD 32 non-SCID PID	2.6 (0.2–17)	27 MUD 10 MMRD TCR ab/CD 19 depleted PBSC	Flu 150 mg/m^2^ Treo 36-42 mg/m^2^ 8 had Melphalan 140 mg/m^2^ for high risk graft rejection 14 had rituximab 1 unconditioned Serotherapy 35 ATG 2 alemtuzumab	16 (range 11–28)	NA	Max grade 2 in 7 patients Only one had grade IV (no conditioning)	1 patient (unconditioned)	96.7	67.7	27% HID: 36% MUD: 28%	10	NA

*aGvHD* acute graft-versus-host disease, *BU* busulfan, *cGvHD* chronic graft-versus-host disease, *CSA* ciclosporin, *ES* engrafted survival, *Flu* fludarabine, *HID* haploidentical donor, *MAC* myeloablative conditioning, *MMF* mycophenolate mofetil, *MMUD* mismatched unrelated donor, *MSD* matched sibling donor, *MUD* matched unrelated donor, *N* neutrophil, *NA* not available, *OS* overall survival, *PID* primary immunodeficiency diseases, *RIC* reduced intensity conditioning, *SCID* severe combined immunodeficiencies, *Treo* treosulfan, *TT* thiotepa, *WAS* Wiskott-Aldrich syndrome

## Pharmacokinetic Studies

Although levels of busulfan have been measured for many years, to target the narrow myeloablative therapeutic window, minimize toxicity from supra-therapeutic levels and avoid sub-myelo-ablation and rejection, it is only recently that the importance of pharmacokinetic monitoring of other agents of the conditioning cocktail has been appreciated.

### Fludarabine Pharmacokinetics

Ivaturi et al. prospectively studied the pharmacokinetics and pharmacodynamics of 133 children undergoing HCT for a variety of disorders with a variety of conditioning regimens but all included fludarabine. Young age and renal impairment were found to lead to an increased exposure. In the setting of malignancy, disease-free survival (DFS) was highest 1 year after HCT in subjects achieving a systemic fludarabine plasma (f-ara-a) cumulative area under the curve (cAUC) greater than 15 mg*hour/L compared to patients with a cAUC less than 15 mg*hour/L (82.6% versus 52.8%, *p* = 0.04) [52]. Further development of model-based dosing may minimize toxicity and maximize efficacy, resulting in superior outcomes for malignant and non-malignant patients.

### Treosulfan Pharmacokinetics

Relatively high variability of treosulfan pharmacokinetics in pediatric patients may raise the need for implementing therapeutic drug monitoring and individual dose adjustment in this group. Vander Stoep et al. and Mohanan et al. recently published the first results of a relationship between the exposure of treosulfan and early toxicity, as well as clinical outcome, in children undergoing conditioning prior to HSCT. In the former study, patients with an AUC > 1650 mg h/L demonstrated a statistically higher incidence of mucosal and skin toxicity than those with an AUC 1350 mg h/L (odds ratio 4.4 and 4.5, respectively). The odds of developing hepato- and neurotoxicity were also higher in the former group, but the difference did not reach statistical significance. No association was found between treosulfan exposure and early clinical outcomes, i.e., engraftment, donor chimerism, acute graft-versus-host disease, treatment-related mortality, and overall survival. PK parameters were shown to be age-dependent, with higher AUC values in younger children (< 1 year old) and corresponding lower treosulfan clearance. A challenge in therapeutic monitoring of treosulfan within conditioning prior to HCT is a very brief course of treatment, consisting of three doses administered on 3 consecutive days. This allows personalization of only the second and third dose of the prodrug unless a test dose is applied prior to starting the actual regimen.

Since pharmacokinetic studies of treosulfan began, it has been assumed that plasma (serum) concentrations of the prodrug are a good representation of the alkylating activity of its epoxy transformers. However, for years, a correlation between treosulfan concentrations in plasma and levels of specific DNA adducts in tissues, for example the bone marrow, or clinical effects, have not been investigated. Therapeutic drug monitoring of not only prodrug but also its active epoxide might be needed. In addition blood pH, body temperature, and intravenous fluid delivery may influence glomerular filtration, tubular reabsorption, and nonenzymatic epoxy transformation of the prodrug [53].

### Serotherapy Levels

It is now well recognized that type of serotherapy, dose and timing in relation to the transplant all have an impact on outcome of transplant in terms of occurrence of GVHD, immune reconstitution importantly in terms of viral reactivation, clearance of infection, and chimerism. Marsh RA et al. collected data from 105 patients to examine the influence of peritransplant alemtuzumab levels on acute GVHD, mixed chimerism, and lymphocyte recovery. Significantly higher levels of aGVHD but higher levels of donor chimerism, lymphocyte counts at D+30 and T cell counts at D+100 were associated with lower alemtuzumab levels at day 0 [54].

In a recent report, the clearance of the active components of the 2 widely used types of ATG (Fresenius/Grafalon and Genzyme) was studied in 38 children with malignant hematological disorders. They found that ATG Fresenius was cleared rapidly and uniformly from the circulation whether they received 60 mg/kg or 45 mg/kg, but there were significant differences in patients who received a high dose of ATG Genzyme (10 mg/kg) who had significantly slower reconstitution for CD3, CD4, and CD8 T cells compared to patients who received a low dose of ATG Genzyme (6–8 mg/kg) or ATG Fresenius [55].

## Stem Cell Source in Non-MAC Conditioning

Historically bone marrow has been the preferred stem cell source for HCT in children due to concerns that peripheral blood stem cell products led to an increased risk of GVHD. In Slatter et al.’s report of 160 PID patients who received uniform conditioning with treosulfan and fludarabine, a higher level of myeloid chimerism was found in recipients of PBSC compared to CB and BM, without an increased risk of grade III/IV acute or chronic GvHD [26]. This is an important finding particularly for patients with diseases where a high level of chimerism is required to achieve complete cure.

## Conclusions

The use of RTC and RIC has been a major paradigm shift in HCT for PID and may have contributed to improved survival through a reduction in early post-HSCT toxicities. Almost certainly, long-term toxicities will be reduced, although further data are required to confirm this. However, the use of antibody-based conditioning regimens is likely to transform the field in the future. The drive for this has been that PID can be completely cured by HCT, and as malignancy is rarely a feature of the disease, toxicity from the curative procedure should be minimized. More recently, newborn screening for severe combined immunodeficiencies has meant that these patients are now being identified by 2–3 weeks of age [56]. Rapid transplantation is preferred, as survival and neurological outcome results are best in patients with no preexisting infection [57, 58]. As gene therapy approaches become mainstream treatment, then a non-toxic conditioning approach followed by an autologous gene-corrected stem cell procedure should almost eliminate short- and long-term treatment-related morbidities for patients with SCID [59, 60]. These conditioning approaches will have to be modified for combined immunodeficiencies and gain-of-function diseases where high-level or complete donor chimerism is required to abolish disease manifestations [61–64]. However, combinations of antibody-based regimens and pharmacokinetically targeted reduced low-toxicity agents may help resolve these issues. The future for patients with PID looks extremely encouraging.
